# Mollugin: A Comprehensive Review of Its Multifaceted Pharmacological Properties and Therapeutic Potential

**DOI:** 10.3390/ijms262412003

**Published:** 2025-12-13

**Authors:** Sandra Ross Olakkengil Shajan, Bushra Zia, Charu Sharma, Sandeep B. Subramanya, Shreesh Ojha

**Affiliations:** 1 Department of Pharmacology and Therapeutics, College of Medicine and Health Science, United Arab Emirates University, Al-Ain P.O. Box 15551, United Arab Emirates; 700046447@uaeu.ac.ae (S.R.O.S.); 700043004@uaeu.ac.ae (B.Z.); 2 Department of Genetics and Genomics, College of Medicine and Health Science, United Arab Emirates University, Al-Ain P.O. Box 15551, United Arab Emirates; charusharma@uaeu.ac.ae; 3 Department of Physiology, College of Medicine and Health Science, United Arab Emirates University, Al-Ain P.O. Box 15551, United Arab Emirates; sandeep.bs@uaeu.ac.ae

**Keywords:** mollugin, anticancer, NF-κB/MAPK signaling, anti-inflammatory, neuroprotection, Nrf2 pathway

## Abstract

The substantial interest in plant-based drugs or plant-derived phytocompounds drives researchers to conduct comprehensive investigations on their therapeutic properties. Mollugin, one of the major active constituents of *Rubia cardifolia*, has been well-studied for its pharmacological properties, demonstrating potent anti-inflammatory properties by suppressing the TAK-1-mediated activation of NF-κB/MAPK and enhancing the Nrf2/HO-1-mediated antioxidant response. It exhibits strong anticancer effects through ferroptosis via IGF2BP3/GPX4 pathways, induces mitochondrial apoptosis, and targets NF-κB, ERK, and PI3K/Akt/mTOR to suppress tumor progression. Mollugin also inhibits JAK2/STAT and PARP1 pathways, suppressing IL-1β expression via the modulation of ZFP91. Moreover, it regulates the MAPK/p38 pathway, promotes neuroprotection, and improves cognitive performance through GLP-1 receptor activation. Mollugin promotes osteogenesis by activating the BMP-2/Smad1/5/8 signaling pathway and downregulates MAPK, Akt, and GSK3β expression, leading to the inhibition of osteoclastogenesis. It overcomes multidrug resistance by downregulating MDR1/P-gp, CREB, NF-κB, and COX-2 through AMPK activation. Its antibacterial effect is mediated by strong binding to FUR, UDP, and IpxB proteins in *Enterobacter xiangfangensis*. Mollugin mitigates *Klebsiella pneumoniae* infection, suppresses adipogenesis without causing cytotoxicity, and protects endothelial cells via the BDNF/TrkB-Akt signaling pathway. Synthetic derivatives of mollugin, such as oxomollugin and azamollugin, have shown enhanced anticancer and anti-inflammatory effects by regulating EGFR, PKM2, TLR4/MyD88/IRAK/TRAF6, and NF-κB/IRF3 pathways with improved solubility and stability. Collectively, these findings emphasize the broad-spectrum activity of mollugin. This review provides a critical interpretation of the mechanistic pathways regulated by mollugin and its derivatives, emphasizing their pharmacological significance and exploring their potential for future translation as multitarget drug candidates.

## 1. Introduction

Over the past decade, there has been a significant increase in the use of plant-based formulations as phytopharmaceuticals, botanical medicine, dietary supplements, or nutraceuticals. They have gained wide public acceptance due to the perception of being natural and safe, with minimal adverse effects over synthetic drugs. The plant-based preparations are common in traditional Chinese medicine (TCM) as well as in Indian Ayurvedic medicine, and for thousands of years they have played an essential role in the prevention and disease treatment [1]. In addition to their popular use as a remedy in chronic diseases, the phytochemicals derived from the plants have gained scientific interest as a source of novel drug discovery. They are now studied for the precise pharmacological rationale and mechanisms observed in experimental models.

In the last few years, numerous plants and plant-derived phytochemicals belonging to the different chemical classes, viz. alkaloids, saponins, terpenoids, etc., have been evaluated for their biological actions, pharmacological properties, and molecular mechanisms, as well as their toxicological potential. The experimental models relied on different kinds of in vivo, in vitro, and in silico studies and evaluated the herbal extracts, different extract fractions, and isolated phytochemicals. Many of the studies are now focusing on evaluating the active constituents, i.e., phytochemicals contributing to the pharmacological effects of the herb. Identifying the active phytoconstituents helps in validating the traditional claim of the particular plant and provides a pharmacophore template for future drug discovery utilizing computational and synthetic approaches.

Among the numerous herbs studied, one of the herbs, known as *Rubia cordifolia* Linn. (Family Rubiaceae), which has been used for decades in different traditional medicines [2]. It is generally known as Qiancao in Chinese and Manjistha in Sanskrit and is officially acknowledged in the Chinese Pharmacopoeia [2]. This plant has been shown to possess numerous pharmacological properties such as antioxidant, anti-inflammatory, anticancer, antihyperglycemic, gastroprotective, neuroprotective, antibacterial, and immunomodulatory effects in experimental studies, demonstrating potential for therapeutic application in various human diseases. The herb has been reported to consist of more than 100 phytochemicals belonging to different classes such as glycosides, polysaccharides, hexapeptides, and triterpenoids [3]. The majority of the pharmacological properties are attributed to the phytoconstituents present in the plant, which contribute to its therapeutic potential. One of most important bioactive agents identified in the plant is mollugin, which has received significant attention for pharmacological evaluation in the past few years [3]. Therefore, this review provides a systematic and critical analysis of the existing evidence of the molecular mechanisms, pharmacological and therapeutic properties of mollugin and its derivatives. It focuses on the documented findings, discusses results from in vitro and in vivo animal models, and provides insights for future translational and clinical studies.

## 2. Pharmacological Activities of Mollugin

*Rubia cardifolia* is a perennial thorny climber that grows widely in the Indian Western Ghats and lower Himalayan ranges. The wet tropical forests of Indonesia, Sri Lanka, and Japan are also home to this plant [4]. The leaves are ovate-lanceolate, measuring 2–10 cm long. The flowers are tiny, fragrant, and appear in either white or greenish yellow. The fruit is small glabrous, has one or two seeds, and when fully grown, it turns dark purple or blackish color. The roots of the *Rubia cardifolia* are a valuable source of medicine; Manjistha is the common name for the root of the plant, which is marketed as Manjith [5].

Manjistadi kashayam is an ancient Ayurvedic herbal extract that mainly relies on Indian madder (*Rubia cardifolia*) as its primary Ayurvedic component. The formulation serves as a treatment for various skin disorders including psoriasis, allergic dermatitis, vaginal ulcers, and chronic skin ulcers. Furthermore, gout, rheumatoid arthritis, and other joint-related conditions can be effectively treated with this medication. *R. cordifolia* also has other well-documented applications in traditional medicine, such as the treatment with fruits for hepatic obstructions, stem for cobra bite and scorpion sting [6], and its roots for amenorrhea, chronic diarrhea, nephrolithiasis, jaundice, and neuromuscular immobility [4].

According to the phytochemical investigations on *R. cordifolia*, its bioactive constituents include triterpenoids, anthraquinone glycosides, naphthoquinones, and compounds based on cyclic hexapeptide and anthracene. Among these, the bioactive compound mollugin, a hydroxy-substituted naphthoquinone, primarily extracted from the roots of *R. cordifolia*, has gained attention for its pharmacological potential [2]. It is isolated and purified from the roots of *R. cordifolia* using ethyl alcohol extract with a preparative high-speed counter current chromatographic method. This technique is performed using petroleum ether at a boiling point of 60–90 °C, with diethyl ether and water as the biphasic solvents [2]. Alternatively, mollugin can be chemically synthesized from 1,4-naphthoquinone as the starting material through a series of reactions. It can be synthesized by reacting triethylamine with methyl 3-(3-methyl-but-2-enyl)-1,4-dioxo-1,4-dihydro-naphthalene-2-carboxylate through the oxa-6π pericyclic reaction [7].

**Figure 1:**
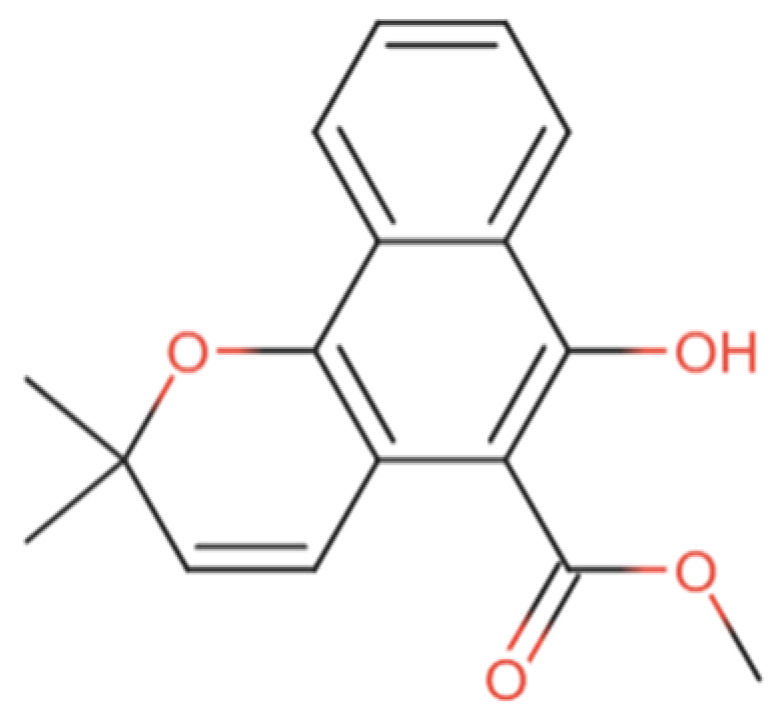
The chemical structure of mollugin.

The chemical structure of mollugin is presented in Figure 1. Mollugin, chemically methyl 6-hydroxy-2,2-dimethylbenzo[h]chromene-5-carboxylate, also known as rubimaillin, is a benzochromene derivative with a dimethyl group at the 2-position, a methoxycarbonyl group at the 5-position, and an OH group at the 6-position [8].

Mollugin demonstrates a broad spectrum of pharmacological properties, including antioxidant, anti-inflammatory, antimutagenic, antiplatelet, anticancer, gastroprotective, vascular-protective, antiulcerative, antiadipogenic, antiresorptive, antimicrobial, neuroprotective, and antiviral properties, notably against the hepatitis B virus [9,10]. Rubiaceous herbs containing mollugin hold great importance in Chinese traditional medicines due to its anticancer, expectorant, antitussive, and blood circulation-promoting properties [11]. In addition, mollugin effectively inhibits platelet aggregation triggered by collagen and arachidonic acid [12]. It also inhibits both acetyl-CoA acetyltransferase 1 (ACAT1) and acetyl-CoA acetyltransferase 2 (ACAT2) enzymes, with a predominant inhibitory effect on the ACAT2 isozyme. This modulation influences cholesterol metabolism and plays a significant role in cardiovascular and metabolic diseases [13]. Among its distinct pharmacological properties, its anticancer effect is more significant, as it involves multiple mechanistic pathways. The pharmacological properties, molecular mechanisms, and therapeutic potential of mollugin are discussed here in this review.

### 2.1. Anticancer Effects of Mollugin

Cancer is a widespread health concern and accounts for one out of every six deaths worldwide [14]. Carcinogenesis is a complex process in which dysregulated cell growth gives rise to malignant tumors that are capable of invading other body organs. Cancer arises from genetic mutations, epigenetic modifications, inherited gene alterations, or environmental factors [15]. Over the past few years, a range of phytochemicals have been investigated for their anticancer efficacy, and several of them have exhibited significant cytotoxic effects. About 50% of approved potent chemotherapeutic agents are obtained from plant-based phytoconstituents such as vincristine, vinblastine, paclitaxel, and camptothecin. These are extensively prescribed and they act therapeutically through the regulation of several mechanisms like microtubule destabilization, cell cycle arrest, topoisomerase enzyme inhibition, and by initiating apoptosis [16,17].

The medicinal potential of these compounds highlights the impact of plant-derived phytoconstituents in cancer therapy. Among emerging bioactive plant compounds, mollugin has achieved notable attention due to its promising anticancer properties through the regulation of multiple mechanisms such as ferroptosis, mitochondrial-mediated apoptosis, and antiproliferative effects. One of the in vitro studies conducted by Wang et al. (2023) on colorectal carcinoma (CRC) cell lines reported that mollugin increases reactive oxygen species (ROS), Fe^2+^, and malondialdehyde (MDA) levels while lowering glutathione (GSH) levels [18]. Consequently, treatment of CRC cells with mollugin results in a significant decrease in the cell viability, which is subsequently blocked by oxytosis inhibitor ferrostatin-1 and enhanced by the ferroptosis inducer erastin. Together, these findings indicate that mollugin inhibits the IGF2BP3/GPX4 (insulin-like growth factor 2 mRNA-binding protein 3/glutathione peroxidase 4) axis and induces ferroptosis in CRC cells, establishing its effective chemotherapeutic potential [18]. These results highlight the ferroptosis mechanism as a strong finding through which mollugin exerts its anticancer effects. Extending the investigation of the ROS-mediated mechanism to an alternative model by Ke et al. (2022) reported that mollugin suppresses the proliferation of HepG2 cells by causing cell cycle arrest [19]. This action is facilitated by excessive ROS generation and the downregulation of cyclin A2 and cyclin-dependent kinase 2 (CDK2) expression, resulting in oxidative stress, DNA damage, and cell cycle interruption in the S phase, finally resulting in cell death. The Western blot analysis reveals a dose-dependent rise in p-H2AX levels, indicating DNA damage in hepatoma cells. These findings indicate that mollugin significantly inhibits tumor development, reduces tumor volume, and notably increases ROS concentration in the tumor tissue [19]. Mollugin downregulates essential cell cycle regulators by altering the expression of CDK2 and cyclin A2 [19]. This multitargeting, caspase-mediated apoptosis and the inhibitory effects on cell division demonstrate its potential anticancer properties.

Apart from its regulatory role in the cell cycle, mollugin induces cell death through well-established apoptosis pathways. In glioblastoma cells, mollugin suppresses cell viability by inducing mitochondrial apoptosis, and the expression of autophagic markers is upregulated. In addition, researchers have identified that mollugin triggers the extracellular signal-regulated kinase (ERK) and PI3 kinase (Phosphoinositide 3-kinases)/AKT/mTOR/p70S6K pathways involved in apoptotic signaling [20]. It also elevates the apoptosis promoting factor Bax and downregulates the antiapoptotic factor Bcl-2, which triggers apoptosis in human ovarian cancer SKOV-3 cells through the translocation of cytochrome C from mitochondria, which in turn promotes the activation of Poly ADP-ribose polymerase (PARP) and caspase 3 [21]. The molecular mechanism through which mollugin exerts anticancer activity involves the activation of caspases, inhibition of cell cycle regulators, and overcoming multidrug resistance. Endoplasmic reticulum-induced stress triggers the upregulation of c-Jun N-terminal kinase and caspase-12. Subsequently, caspases 3 and 9 are activated by a Bcl-xL-controlled mechanism via a mitochondrial-dependent pathway, which in turn activates caspases 7 and 8, ultimately leading to apoptosis and cytotoxic effects on Jurkat T cells [22]. Overall, mechanistic insights demonstrate that mollugin promotes anticancer activity through the consistent activation of mitochondrial and caspase-dependent apoptosis.

Expanding the spectrum of targeted intracellular pathways, mollugin attenuates proliferation and induces caspase-mediated cell death by downregulating the production of the FAS receptor gene via the ERBB2/Akt/SREBP-1c molecular pathway in human epidermal growth factor receptor 2 (HER2) overexpressed tumor cells. Based on these results, Do et al. (2013) conclude that mollugin is an emerging regulator of the ERBB2 signaling pathway in malignant cells, and it has the potential to manage ovarian and breast carcinoma [23].

Further, mollugin has been found to be an effective active component against Nasopharyngeal carcinoma (NPC). Annexin V-FITC/PI double fluorescence labeling and MTT assay reveal that mollugin suppresses NPC cell growth and promotes apoptosis. Further investigations confirm that the regulation of Survivin and XIAP expression is associated with the molecular mechanism underlying the protective effects of mollugin against NPC [24]. Regarding intracellular signaling mediating inflammation, mollugin substantially and dose-dependently downregulates the expression of an NF-κB gene activated by tumor necrosis factor-alpha (TNF-α). It also inhibits TNF-α-mediated phosphorylation and nuclear migration of p65, along with the phosphorylation and protein degradation of the IκBα antagonist. Furthermore, mollugin pretreatment inhibits TNF-α-triggered activation of nuclear factor kappa B and regulates genes like matrix metalloproteinase-9, CD54, vascular endothelial growth factor, Bcl-2, cIAP-1, survivin, cyclooxygenase 2 (COX-2), cyclin D1, and the proto-oncogene c-Myc. Mollugin also inhibits the proliferation of tumor grafts derived from HeLa cell lines. These in vivo findings suggest that mollugin could be a promising candidate for chemotherapy by therapeutically targeting the NF-κB pathway [25] but extensive studies are still lacking.

Notably, mollugin not only regulates apoptosis and proliferation, but also counteracts the chemotherapy resistance mechanism. Tran et al. (2013) found that AMP-activated protein kinase (AMPK) is activated by mollugin, which in turn suppresses multidrug resistance (MDR1) promoter activity and thereby downregulates protein expression [26]. Mollugin also attenuates the phosphorylation of NF-κB transcription and cAMP response element-binding protein (CREB), lowers MDR1 expression, and also suppresses the expression of COX-2. These findings imply that mollugin suppresses P-glycoprotein (P-gp) expression via blocking the COX-2 and NF-κB signaling pathway, as well as downregulating CRE transcriptional activity by activating AMPK. The inhibition of P-gp by mollugin may help to overcome multidrug resistance in chemotherapy [26].

Despite its broad anticancer properties, the physiochemical instability and poor pharmacokinetic profile limit the efficacy of mollugin. This highlights the need for structural modifications, which has led to the chemical synthesis of mollugin derivatives. Mollugin derivatives have provided further mechanical insights. For instance, FFJ-3, a chemically modified derivative of mollugin with a para-nitrobenzyl ether substituent covalently attached to the OH group on the chromone ring, exhibits enhanced anticancer efficacy against three human cancer cell lines: HepG2, human breast adenocarcinoma MCF-7, and A549. FFJ-3 downregulates pyruvate kinase M2 (PKM2), attenuates PI3 kinase/Akt signaling, induces caspase 3 activation, and modifies the Bcl-2/Bax ratio, thereby facilitating apoptosis in tumor cells [27]. With this effective demonstration of anticancer activity by FFJ-3, a similar study was carried out by the same group of researchers, in which a novel naphthoquinone mollugin derivative, referred to as FFJ-5, was studied for its anticancer properties on A549 and HepG2 cell lines. The study reported that FFJ-5 downregulates PKM2, the epidermal growth factor receptor (EGFR), and the Akt-mediated signaling pathway, and also reduces the production of ATP in cancer cells, leading to energy starvation. FFJ-5 also decreases mitochondrial membrane potential and lowers intracellular pH. Moreover, G2/M phase cell cycle arrest was observed in HepG2 and A549 cell lines [28].

Several synthetic triazole derivatives of mollugin were synthesized and tested for cytotoxicity, and those derivatives containing methoxy groups showed better cytotoxic effects. According to the structural activity relationship (SAR) study, electron releasing groups such as the OH group, OCH_3_ group, and alcoholic hydroxyl groups are necessary to maintain the cytotoxic activity in the mollugin derivatives [29]. Taken together, the findings on derivatives further support that chemical modification enhances stability and potency.

**Figure 2:**
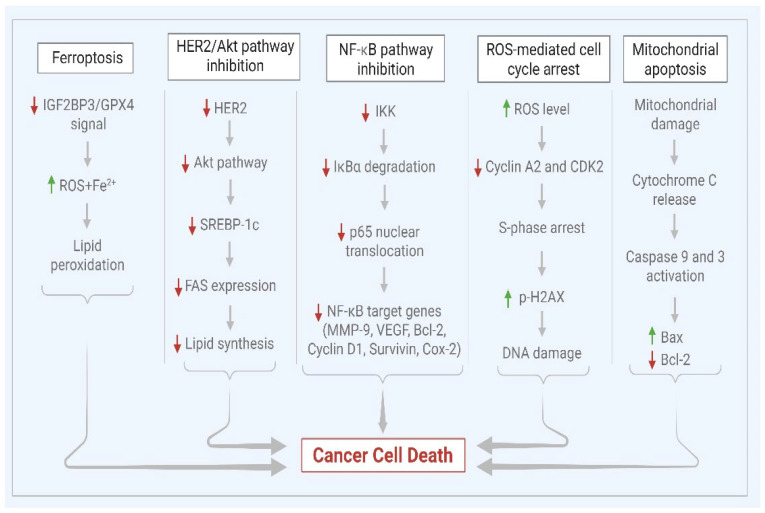
Mollugin-Mediated Anticancer Mechanisms.

Mollugin demonstrates multitargeting properties that reveal the therapeutic potency by inducing cell death and overcoming resistance. Collectively, these findings emphasize the potential of mollugin as a promising anticancer agent. However, mollugin selectively and competitively inhibits the CYP1A2 liver enzyme, with a low IC_50_ value of 1.03 µM [30], and may exhibit potent anticancer properties by preventing the activation of procarcinogens. Since CYP1A2 is responsible for the metabolism of certain clinically significant drugs like theophylline and clozapine, mollugin may cause drug–drug interactions by inhibiting CYP1A2 enzyme activity. Therefore, further studies are required to clarify the scope and therapeutic impact of these interactions to promote safe clinical implementation. Overall, in vitro studies provide substantial evidence for the anticancer properties of mollugin through various mechanistic pathways, whereas there are limited data from in vivo studies, emphasizing the need for broader validation. The anticancer properties of mollugin are depicted in Figure 2.

### 2.2. Anti-Inflammatory Effects of Mollugin

Inflammation is the body’s protective mechanism by which the immune system recognizes foreign invaders and defends against pathogens to eliminate harmful stimuli [31]. It can be acute or chronic, as determined by the trigger and duration. Inflammation is crucial for host defensive response, but it can become detrimental when the inflammatory response remains for a long duration [32]. Acute inflammation can often be resolved with suitable intervention, but in many cases the sustained release of pro-inflammatory cytokine, cytokines, and chemokines damages healthy cells and causes inflammation, leading to conditions such as cardiovascular diseases, neurodegenerative disorders, rheumatoid arthritis, and certain cancers. Thus, targeting inflammation without disturbing normal cellular processes remains a challenge [31,33].

Plant-based bioactive compounds have gained wide acceptance in managing inflammation due to their potent anti-inflammatory properties and high safety margins [34]. Secondary metabolites such as flavonoids, terpenoids, and alkaloids have been proven to regulate inflammatory signaling pathways, such as mitogen-activated protein kinase (MAPK), NF-κB, and nuclear factor erythroid 2-related factor 2 (Nrf2). By modifying these pathways, well known phytoconstituents such as curcumin, resveratrol, and quercetin effectively mitigate inflammation with fewer side effects compared to synthetic drugs. Among various phytochemicals, mollugin is identified as a promising compound because of its multi-pathway modulation in inflammation [35].

Early in vitro investigation by Zhu et al. (2013) [36] demonstrates that mollugin mitigates inflammation when RAW264.7 macrophages are exposed to lipopolysaccharides. It substantially reduces the levels of inducible nitric oxide synthase (iNOS), nitric oxide (NO), interleukin-1 beta (IL-1β), and interleukin-6 (IL-6). However, mollugin does not reduce the levels of TNF-α nor inhibit the breakdown of IκB-α. Instead, it increases the phosphorylation of p65, thereby stimulating the NF-κB pathway. Mollugin did not inhibit ERK1/ERK2, P38, or c-Jun N terminal kinase 1/2 (JNK 1/2) of the MAPK pathway, while it suppresses the activation of janus kinase 2 (JAK2) and signal transducer and activator of transcription 1/3 (STAT1/3). Based on these findings and in silico studies, it is concluded that mollugin interacts with JAK2 in a comparable manner to AG490 (JAK2 inhibitor). Therefore, mollugin may act as a potential JAK2 kinase inhibitor, selectively targeting the JAK2–STAT signaling pathway without affecting other inflammatory pathways [36]. These in vitro findings strongly support that mollugins exert anti-inflammatory activity through JAK2–STAT inhibition.

In 2020, Li et al. conducted an in vivo experiment inducing ulcerative colitis in animals using a 3% dextran sodium sulfate (DSS) solution and reported that mollugin lowers the expression of the pro-inflammatory cytokines, Interlukin-1β and TNF-α, when administered at a dose of 20 and 40 mg/kg. Mollugin treatment markedly ameliorates the severity of DSS-induced ulcerative colitis. Treated animals exhibit a significantly lower disease activity index, reduced colon damage, and a pronounced recovery in body weight compared to untreated controls. Mechanistically, mollugin downregulates toll-like receptor 4 (TLR4) expression in colonic tissue. Collectively, these findings posit mollugin as a potential therapeutic candidate for ulcerative colitis [37]. This provides strong in vivo evidence for its anti-inflammatory effects.

Mollugin derivatives have provided additional insights; Zhang et al. (2022) synthesized 23 new mollugin derivatives to evaluate their anti-inflammatory properties, primarily to determine whether the newly synthesized compounds can inhibit NF-κB activity [38]. Out of all 23 compounds, 2 compounds showed potent anti-inflammatory effects. One of the mollugin derivatives, referred to as compound 6d, with a bicyclic structure where the R3 group is a 1,8 naphthyridine ring, reports promising effects with decreased levels of p65 and also inhibits the NF-κB pathway. The reported IC_50_ value is 3.81 µM and the compound is found safe with few cytotoxic effects. Another derivative identified as compound 4f, where R3 group is a phenyl ring with fluorine at para position, exhibits an electron-withdrawing effect due to the presence of fluorine. This compound shows significant anti-inflammatory effect in vivo, with an inflammation rate of 83.0%, which is more effective than ibuprofen and mesalazine. Newly synthesized mollugin derivatives exhibit remarkable ADMET properties, suggesting that these could serve as potential anti-inflammatory agents [38], although comparative in vivo results remain limited.

Liu et al. (2023) reported that mollugin inhibits the upstream regulation of IKKα/β, MAPK signaling cascade, and TAK1 phosphorylation, which ultimately inhibit the NF-κB- and MAPK-mediated inflammatory pathways [39]. It also stimulates the Nrf2 antioxidant pathway, which lowers ROS levels. Cellular thermal shift assay (CETSA) and molecular docking studies point to the effective binding of mollugin to TAK1 and KEAP 1 [39].

In a murine model of cecal ligation and puncture (CLP)-induced sepsis, mollugin attenuates inflammatory damage to the liver and lung tissue, likely by the downregulation of pro-inflammatory cytokine levels. Collectively, these findings suggest that mollugin has a dual effect on reducing sepsis by attenuating TAK1-mediated NF-κB/MAPK signaling and promotes the activation of the Nrf2 pathway, suggesting that it may be an ideal therapeutic candidate for treating sepsis [39]. Together, the evidence across in vivo and in vitro models suggests that mollugin exhibits anti-inflammatory properties primarily by targeting NF-Κb and Nrf2 pathways.

Molecular docking results indicate that mollugin may act similarly to p38 MAPK or PARP1 inhibitors, as it binds to the same sites where SB 203580 (p38 MAPK inhibitor) and Olaparib (PARP 1 inhibitor) interact. Mollugin downregulates the expression of inflammatory marker arginase-1 and inhibits the activation of the p38 MAPK pathway in immune cells. It also downregulates the expression of PARP1 and inhibits the production of IL-4 and IL-5. Through the modulation of the T helper type 2 (Th2) immune response, mollugin minimizes the severity of allergic reactions in the lungs [40].

In 2024, Gao et al. investigated the anti-inflammatory properties of several mollugin derivatives in an in vitro model. One of the mollugin derivatives with the quimolin-6-yl group, referred to as compound 5k, demonstrates an improved anti-inflammatory action (81.77%) in comparison to other conventional anti-inflammatory medications. Molecular docking studies also prove that compound 5k inhibits the p65 subunit of NF-κB. In silico ADMET prediction indicates that compound 5k has improved pharmacokinetic characteristics and may be a potential candidate for development as an anti-inflammatory drug; however, further in vivo validation is required to assess translational potential [41].

**Figure 3:**
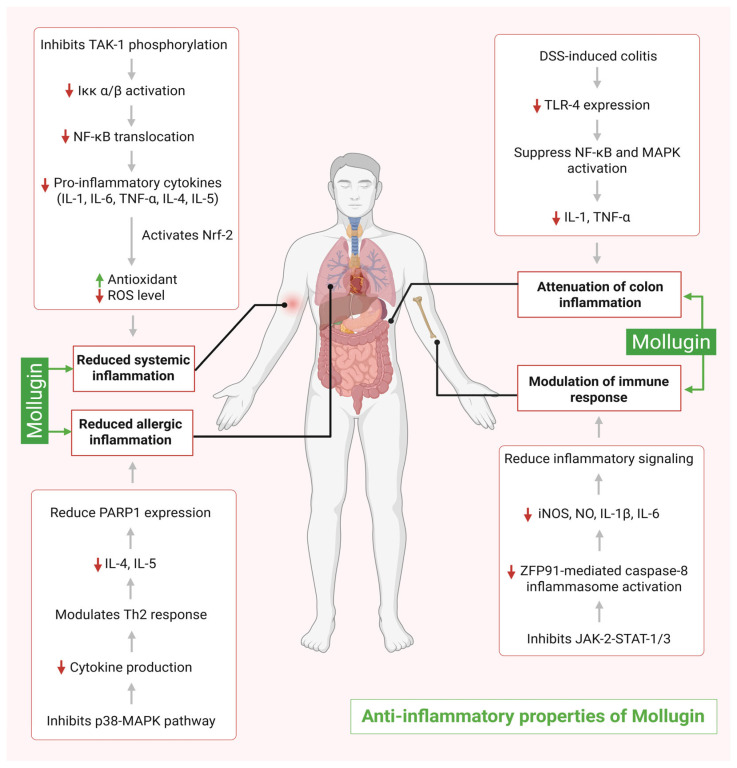
Mollugin-mediated anti-inflammatory mechanisms.

A recent study by Lei et al. in 2025 evaluates the anti-inflammatory effects of mollugin using in vitro and in vivo experimental setups. Mollugin attenuates inflammation by downregulating zinc finger protein 91 (ZFP91) in macrophages, consequently inhibiting the release of the key inflammatory cytokine IL-1β. The caspase-8 inflammasome complex becomes less active as ZFP91 levels go down. The study also shows that mollugin blocks k63-linked ubiquitination of pro-interleukin-1β which serves as the necessary process to activate IL-1β. Research utilizing mice models demonstrates that mollugin can improve gut flora and mitigate colitis and peritonitis [42]. Overall, mollugin emerges as a promising candidate by selectively inhibiting major signaling pathways such as JAK2, STAT, NF-κB, MAPK, and Nrf2. The overall findings from in vitro and in vivo studies are strong and consistent; however, further in vivo studies are warranted to assess the anti-inflammatory properties of mollugin derivatives. The development of mollugin derivates with enhanced efficacy, potency, and favorable pharmacokinetic profiles support its approval as an efficient and safer alternative. Its ability to modulate the inflammasome and enhance the gut microbiota together supports its therapeutic value as an anti-inflammatory agent. Further research is needed to undertake detailed mechanistic validation of mollugin. Figure 3 illustrates the anti-inflammatory properties of mollugin.

### 2.3. Neuroprotective Effects of Mollugin

Neurons are the fundamental units of the nervous system, involved in receiving and transmitting signals throughout the body via action potentials and neurotransmitters [43]. Depending on the severity of the damage, a neural insult can cause anything from a mild sensory deficiency to a severe traumatic injury by interfering with neuronal pathways [44]. Such nerve damage can lead to conditions such as diabetic neuropathy, degenerative neurological disorders, autoimmune diseases, infectious neuropathies, and severe nerve impairment, which can lead to traumatic injuries. Neuroprotective agents are therefore essential, as they protect neuronal structure, prevent damage, and restore degenerative processes [45].

Natural bioactive compounds extracted from herbs promote neuroprotection and prevent the development of neuronal degeneration by modulating critical pathways of nerve damaging mechanisms involving oxidative stress, neuroinflammation [46], and apoptosis, highlighting their role as neuroprotective agents [47]. Among various bioactive compounds, mollugin stands out as a potent neuroprotective agent due to its well-documented anti-inflammatory properties and its proven ability to inhibit the release of cytokines like TNF-α, IL-6 as well as pro-inflammatory enzymes such as COX-2 and iNOS.

An in vitro study conducted by Jeong et al. (2011) reports that mollugin demonstrates strong neuroprotective effects on mouse hippocampal HT22 cells against ROS production and neurotoxicity [9]. Its anti-inflammatory properties are further supported by its inhibition of inflammatory mediators, including cytokines (TNF-α and IL-6) and pro-inflammatory enzymes (COX-2 and iNOS). In addition, mollugin promotes heme oxygenase-1 expression in HT22 and BV2 cells supporting its neuroprotective and anti-inflammatory properties. These results demonstrate that mollugin promotes Nrf2 nuclear translocation and modulates the p38 MAPK pathway in microglial (BV2) and neuronal (HT22) cell lines. This dual action indicates that mollugin activates the Nrf2/HO-1 axis and p-38 MAPK pathways, providing strong mechanistic evidence for its role in mitigating the neuroinflammatory processes underlying neurodegenerative diseases [9].

**Figure 4:**
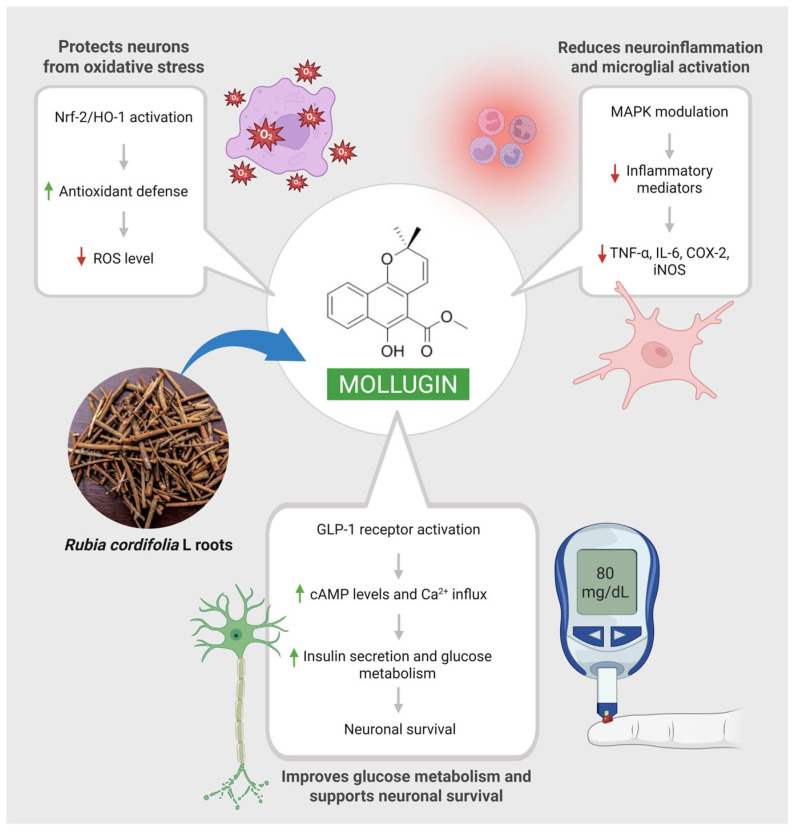
Mollugin-mediated neuroprotective mechanisms.

Furthermore, an in vivo mice model of type 2 diabetes (T2DM) supports its neuroprotective role by showing a notable improvement in cognitive impairment. It binds to glucagon like peptide 1 receptor (GLP-1R), elevates cAMP levels, enhances insulin secretion, and increases Ca^2+^ influx in the β-TC-6 cell line. Moreover, mollugin alleviates neuronal cell damage, reduces PIK3CA, AKT1, and MAPK mRNA expression in the brain cortex tissue, and shortens the latency to the platform in the Morris water maze test performed in T2DM mice [48]. All these results demand further in-depth exploration of mollugin which could establish it as a promising therapeutic agent for neurogenerative diseases. Findings from this study demonstrate that mollugin promotes cognitive performance through the regulation of the GLP-1R and PI3K/Akt signaling pathways. Still, further long-term in vivo studies are needed to establish reproducibility and clinical relevance. The neuroprotective role of mollugin is demonstrated in Figure 4.

### 2.4. Effects of Mollugin in Bone Regeneration and Resorption Suppression

Bone resorption is a common feature of osteoporosis, which commonly occurs in age-related cases or in post-menopausal women, causing a decrease in bone density and making bones more prone to fractures [49]. Phytoestrogens derived from plants have shown significant effects in treating osteoporosis by functioning similarly to mammalian estrogen. For example, soy isoflavones positively impact bone mineral density and provide structural integrity in post-menopausal women [50]. Moreover, isoflavones particularly bind to the estrogen receptor beta (ERβ), mimicking the action of estrogen in bones, while simultaneously inhibiting estrogen effects in other organs like the breast, potentially mitigating the undesirable consequences of traditional estrogen therapy [51].

Moon et al. (2017) examined the anti-osteoporotic activity of mollugin in a zebrafish model and found that mollugin is effective in managing bone degradation and promoting bone regeneration [52]. This effect is achieved by modulating Bone Morphogenetic Protein-2 (BMP-2), which plays a major role in promoting the differentiation of progenitor cells into osteoblasts and is involved in bone formation and in the prevention of osteoporosis progression. Mollugin stimulates BMP-2, which sends signals to activate Smad proteins; these phosphorylated Smads then upregulate the genes necessary for bone formation, significantly improving osteogenesis [52]. Mollugin not only activates BMP-2, but also stimulates bone formation through the modulation of the p-38/smad1/5/8 signaling pathway. This signaling improves osteoblast survival and regulates osteoblastic activity.

Another in vitro study by Baek et al. (2015) evaluated the influence of mollugin and revealed that it suppresses the differentiation of precursor bone cells into osteoclasts and also slows down the bone activity of mature osteoclasts responsible for bone resorption [53]. This impact on osteoclastogenesis is associated with the modulation of key proteins, such as c-Fos and NFATc1, mediated through MAPK, Akt, and GSK3β pathway. Additionally, it also downregulates the expression of osteoclast-specific genes like cathepsin K, tartrate-resistant acid phosphatase (TRAP), and osteoclast-associated receptor (OSCAR), suggesting that mollugin can prevent bone loss [53].

Taken together, both studies demonstrate that mollugin has the potential to prevent bone resorption and promote bone formation, and therefore it could be a potential therapeutic candidate for the treatment of osteoporosis, rheumatoid arthritis, and other bone diseases. Overall, this evidence is moderate in strength and requires additional validation in different models to establish clinical potential. However, more investigation is necessary to compare mollugin with standard drugs such as antiresorptive medications like bisphosphonates, anabolic agents like teriparatide, or with estrogen therapy to determine whether mollugin can be used as an adjuvant or combination therapy for treating bone-related disorders.

### 2.5. Effects of Mollugin in Multidrug Resistance

Multidrug resistance is one of the major challenges in cancer therapy, as it significantly decreases the therapeutic effectiveness. The MDR1 gene encodes P-gp, an ATP-dependent transporter, which serves as a drug efflux pump that expels chemotherapeutic drugs from malignant cells, thereby lowering their intracellular concentration and contributing to multidrug resistance [54]. Mollugin emerges as a promising inhibitor of the MDR1 gene due to its well-known inhibitory effect on NF-κB and CREB transcription factors, which normally promote MDR1 expression leading to drug resistance. Mollugin tested in the MCF-7/adr cell line inhibits the activation of the MDR1 gene in a dose-dependent manner. The downregulation of the MDR1 gene is indicated by the accumulation of Rhodamine-123, a functional assay to measure the P-gp level. Mechanistically, mollugin phosphorylates AMPK, which downregulates the CREB and NF-κB pathway, reducing MDR1 expression. Mollugin also inhibits COX-2 expression, further confirming the suppression of the MDR1 gene. Collectively, these in vitro studies suggest that mollugin provides multidrug resistance reversal through the regulation of the AMPK/NF-κB/COX-2 signaling pathway. These effects may help cells to overcome drug resistance during mollugin treatment [26].

### 2.6. Antimicrobial Effects of Mollugin

Apart from overcoming multidrug resistance, mollugin possesses strong antibacterial activity which expands its therapeutic applications. The rapid rise in antimicrobial resistance necessitates effective medications that overcome resistance and precisely target specific bacterial strains. *Enterobacter xiangfangensis*, a Gram-negative novel pathogenic bacterium from the Enterobacter group, causes UTI infections, lung infections, and septicemia [55]. This pathogen is resistant to a broad spectrum of antibiotics, and currently there is no single approved medication for the specific target, highlighting the demand for novel treatment strategies.

Traditional drug discovery approaches are expensive and time-consuming; these shortcomings are addressed by improved technologies like computational approaches. Almuhayawi et al. (2022) used pan-genome and subtractive genome approaches to find out possible therapeutic drug targets for treating *E. xiangfangensis* [56]. The study identifies 177 non-human homologous proteins, out of which 20 are virulence-associated proteins. Since focusing on cytoplasmic proteins for drug development, researchers narrow down three cytoplasmic proteins—FUR, UDP and IpxB—as promising targets. Through molecular docking analysis, 5000 chemical compounds were screened and 5 natural compounds, namely adenine, mollugin, xanthohumol C, sakuranetin, and toosendanin, with strong binding affinity and favorable docking scores were identified. Mollugin, one of these five compounds, also complies with Lipinski’s rule of five, indicating its potential as a medicinal compound for the treatment of *Enterobacter xiangfangensis* infection [56]. Supporting its potential, Idhayadhulla et al. (2014) find that a carboxylic acid derivative of mollugin, referred as compound 3, exhibits antibacterial property comparable to ciprofloxacin against the *S. aureus* bacteria [57]. Furthermore, 3,4-dihydromollugin shows stronger antioxidant activity than a synthetic antioxidant, 2,6-di-tert-butyl-4-methylphenol (BHT). These findings emphasize that mollugin derivatives are promising molecules for the development of effective antibacterial and antioxidant agents [57].

*Klebsiella pneumoniae* is a prevalent Gram-negative bacterium in the Enteric bacteria family which colonizes in the GI tract, nasal passages, and other segments of the digestive system. It is responsible for various conditions such as pneumonia, urinary infection, septicemia, and hepatic abscess and seriously impacts people with compromised immune systems. A significant proportion of people acquire these infections from health care facilities and this is classified as nosocomial infection [58]. The resistance to carbapenem acquired by this strain raises the concern of multidrug resistance, drawing significant attention to the development of medication that can combat MDR [59]. A study reported in 2019 by Zhang et al. revealed that mollugin treatment in *Klebsiella pneumonia*-infected rats shows lower water content in the lungs [60]. Treatment also reduces neutrophil and WBC counts, suppresses inflammatory cytokines levels, and downregulates the expression of NF-κB, p-MAPK, p-JNK, as well as p-ERK proteins in the lung tissues compared to pneumonia control groups, demonstrating the mitigation of the *Klebsiella pneumoniae* infection in rat lung tissue [60]. Notably, mollugin has only a marginal effect on inhibiting plasmodial activity, with an IC_50_ below 1 µg/mL [61]. However, it also shows strong antifungal and antibacterial effects against plant-based pathogens [62]. Collectively, in silico, in vitro, and in vivo studies indicate that mollugin might have a strong broad-spectrum antimicrobial potential, making it an effective candidate regardless of its weak antiplasmodial activity.

### 2.7. Antiadipogenic Effects of Mollugin

Obesity has become a global health concern due to its associations with a number of conditions, including type II diabetes, hypertension, coronary artery disease, osteoarthritis, sleep apnoea, cancer, and occupational dysfunction [63]. Obesity results from an imbalance between energy consumption and expenditure, which results in increased adipose tissue mass from both hyperplasia and hypertrophy. In the adipogenic process, preadipocytes proliferate and differentiate into mature adipocytes. However, the amount of lipids generated in the adipocytes mostly influences the growth of fat cells [64].

For the first time, Jun et al. (2011) investigated the adipocyte inhibitory effect of mollugin in an in vitro model using 3T3-L1 cells [65]. They found that a high dose of 40–60 µM induces apoptotic events, observed by mitochondrial damage and the activation of caspase 9,3 and 7 leading to breakdown of PARP protein. Mollugin does not affect cell viability at a concentration of 20 µM; however, it inhibits preadipocyte differentiation into mature adipocytes. Furthermore, from day 0 to day 2, mollugin was highly effective in the early stages of adipocyte formation, while its effectiveness decreased from day 4 to 6, suggesting that mollugin can suppress adipocyte development in a dose-dependent manner both with and without causing cytotoxicity [65]. These findings provide a strong mechanistical understanding of the role of mollugin in its antiadipogenic effect and highlight the need to explore its translational potential.

### 2.8. Effects of Mollugin in Ulcerative Colitis

Individuals who are genetically predisposed and have an impaired immune system may mistakenly attack the gastrointestinal tract, leading to inflammation that contributes to inflammatory bowel disease (IBD). Ulcerative colitis (UC) and Crohn’s disease are the primary chronic conditions recognized under IBD [66]. Ulcerative colitis impairs the regulation of epithelial barrier integrity and affects the protective innermost lining of the colon and rectum. The inflammation observed in UC is limited to mucosa, spreading superficially and evenly across the affected GI area, leading to an inflammatory response. In contrast to Crohn’s disease, ulcerative colitis is generally not regarded as a progressive condition; however, it may have additional long-term effects that are independent of progressive inflammatory damage [67].

Anti-inflammatory and immunomodulators are key drugs in managing ulcerative colitis. For the first time, Kim et al. in 2009 synthesized mollugin analogs to compare their therapeutic potential against inflammation in an in vitro model. Compound M1 represents the natural mollugin structure; M2, M3, and M4 are mollugin derivates. In M2, the 2,3-dihydrofuran ring moiety is replaced by the dioxolane ring, in M3 a six-membered cyclic ether is attached to the dioxane ring, and in M4 a long chain aliphatic side chain is attached. In ulcerative colitis, the adhesion process is critical because immune cells bind to the intestinal lining, triggering an immune response that initiates inflammation. The study proves that these derivatives inhibit the adhesion of U937 immune cells to human colon cell line (HT-29), thus preventing early inflammatory events.

Among the four derivatives, mollugin (M1) exhibits potent anti-inflammatory property by significantly downregulating the expressions of inflammatory cytokines, such as MCP-1, IL-8, and ICAM-1, while inhibiting the transcription of NF-κB, a key regulator of inflammation. Furthermore, mollugin in combination with Pyrrolidium dithiocarbamate (PDTC), a potent and selective inhibitor of NF-κB, demonstrates a synergistic anti-inflammatory effect. By inhibiting both 5-lipoxygenase and cyclooxygenase pathways, mollugin demonstrates superior efficacy compared to 5-ASA, the standard drug for IBD. These data suggest that mollugin and mollugin derivatives provide mechanistic effects through NF-κB and lipoxygenase pathways. Supporting this, recent in silico interaction studies by Xu et al. (2024) reported that the Qin-Yu-Qing-Chang decoction, which includes mollugin as a key constituent, acts as a potent PPAR-γ agonist [68]. This activation alleviates the inflammatory response, restores intestinal barrier integrity, and mitigates pathogenic intestinal bacterial populations in a murine model of ulcerative colitis [68]. Through the inhibition of multiple inflammatory pathways, mollugin enhances the effectiveness as a potent anti-inflammatory agent and could alleviate inflammatory bowel diseases such ulcerative colitis [10]. Taken together, in vitro studies on mollugin derivatives strongly support the contribution of the NF-κB and lipoxygenase pathways, while in vivo studies confirm PPAR-γ upregulation and intestinal barrier protection mediated by mollugin in ulcerative colitis.

### 2.9. Effects of Mollugin in Neurovascular Protection

Stroke is a life-threatening condition of the brain, mainly categorized as ischemic and hemorrhagic, and is associated with neurological dysfunction and increased risk of death [69]. A clinical pathophysiological feature of stroke is the disruption of the blood–brain barrier (BBB), which affects the brain homeostasis leading to edema, inflammation, and neuron damage [70]. Understanding these complications leads to explorations of new therapeutic targets.

A very recent study by Jia et al. in 2025 explores the neuroprotective effect of mollugin, using a stroke-like condition mimicked by the disruption of the blood–brain barrier, which causes the impairment of tight junctions that protect the brain microvascular endothelial cells. Using the oxygen–glucose deprivation/reperfusion (OGD/R) model to establish the condition, the study found that mollugin promotes cell survival, reduces apoptosis, and lowers the levels of cleaved caspase 3 in brain microvascular endothelial cells. Mollugin also preserves the structural integrity of the BBB by preventing the loss of barrier-forming proteins such as ZO-1 and Claudin 5. Furthermore, mollugin exerts its protective effects by the activation of the BDNF/TrkB and Akt signaling pathway. When this signaling pathway is particularly inhibited, the protective effect of mollugin is no longer observed, confirming that its action depends on this signaling pathway [71]. Overall, mollugin emerges as a highly promising neuroprotective candidate by activating the BDNF/TrkB and Akt signaling pathways and preserving the structural integrity of the blood–brain barrier, thereby emphasizing its therapeutic promise in neurovascular protection and management. However, data are limited and require further validation in additional stroke models.

### 2.10. Effects of Mollugin in Pain Modulation

Pain is a common symptom which significantly affects the quality of life. Analgesics available today are often less effective and can produce adverse effects. A study conducted by Yan et al. evaluated the analgesic effect of mollugin, also known as rubimaillin, both in vitro and in vivo. In cell line studies, lipopolysaccharides (LPS) (1 μg/mL) induced inflammation in RAW 264.7 mouse cell line, while rubimaillin treatment significantly downregulating iNOS and COX-2 expressions and notably inhibited nitric oxide levels, suggesting analgesic and anti-inflammatory properties. The in vivo experimental method employed acetic acid to induce visceral pain, whereas neuropathic and inflammatory pain was induced by formalin. The research revealed that rubimaillin decreased the pain-associated writhing response in animals, reduced serum cAMP expression, and also suppressed PEG2 levels, which are associated with nociceptive pathways. These findings indicate that rubimaillin has the potential to emerge as a natural non-opioid analgesic agent, and further investigation is required for a better understanding of rubimaillin on neuropathic pain and inflammation [72].

**Figure 5:**
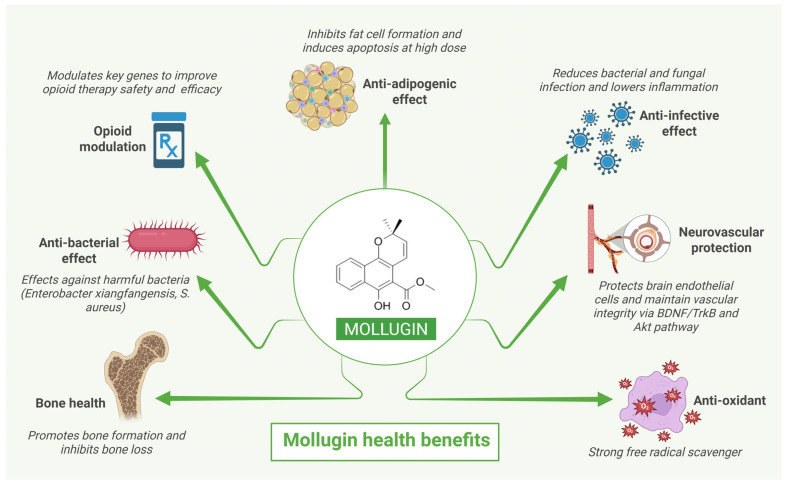
Mollugin-mediated health benefits.

In silico pharmacogenomics analysis, conducted recently in 2024, identified 10 chemicals that interact with the key genes involved in opioid response and pain management. The principal genes involved are ABCB1, BCL2, CYP1A2, KCNH2, PTGS2, and DRD2 which may potentially regulate the physiological effects of opioids in the body. Additionally, PTGS2 and DRD2 could also modulate pain-processing pathways. Out of the ten chemical compounds analyzed, rubimaillin was found to interact with two of the previously mentioned genes. Protein chemical interaction analysis confirmed that rubimaillin interacts with the ABCB1 gene; the other gene involved in the interaction remains uncertain. The ABCB1 gene encodes P-gp, which is found in the blood–brain barrier and pumps drugs and harmful compounds out of brain. This gene modulation may reduce drug efficacy or increase drug accumulation in brain. The influence of rubimaillin on ABCB1 is unclear; the inhibition of ABCB1 might lead to an increased opioid concentration in the brain which also raises concerns about adverse effects. Conversely, the upregulation of ABCB1 may reduce drug efficacy. Understanding this interaction will provide further insight into personalized pain management treatment. Determining the influence of rubimaillin is crucial to understanding whether it can be offered as an alternative or can be an adjunct that can modulate opioid efficacy or reduce adverse effects. More clinical validation is needed to identify the influence of rubimaillin on the ABCB1 gene [73]. The health benefits of mollugin are summarized in Figure 5.

## 3. Development of Mollugin Derivatives and Their Role

The synthesis and optimization of mollugin derivatives are employed to address physicochemical instability and improve poor pharmacokinetic profiles, thereby enhancing their specificity and efficacy in targeting various disease conditions. Nakajima et al. first studied the anti-inflammatory properties of mollugin and its derivatives in 2015. According to their findings, mollugin shows a limited inhibitory effect on the inflammatory signaling pathway, mildly reducing inflammation and the production of iNOS and IFN-β gene expression. However, its oxidized derivative, oxomollugin, exhibits a potent anti-inflammatory effect by inhibiting the phosphorylation and degradation of IκB-α and significantly lowers the production of the IFN-β gene in lipopolysaccharide-induced inflammation [74]. Later, in 2016, it was reported that azamollugin, a nitro derivative of oxomollugin, demonstrates an even stronger inhibitory effect with an IC_50_ value of 0.34 µM compared to oxomollugin with IC_50_ of 1.3 µM, indicating that azamollugin is significantly more potent than oxomollugin [75]. Structural modification of mollugin promotes its anti-inflammatory properties, and azamollugin emerges as the most potent derivative among the compounds tested.

Based on these studies, Nakajima et al. in 2024 further explored the anti-inflammatory and molecular mechanisms of oxomollugin on the RAW 264.7 immune cell line. From this study, it was found that mollugin derivatives inhibit the TLR4 signaling pathway, which mediates LPS-triggered inflammation. Oxomollugin weakens the binding between MyD88 and IRAK4, delaying the phosphorylation of IRAK1 which contributes to the suppression of the inflammatory signaling cascade. Furthermore, oxomollugin inhibits TRAF6 ubiquitination, which is essential for the activation of NF-κB, and also blocks the interaction between TAK1 and TAB2, affecting the inflammatory reaction in the later phase. It also reduces the mRNA expressions of cytokines, thereby preventing NF-κB-associated inflammatory reactions [76].

Similarly, azamollugin possesses strong anti-inflammatory properties by preventing nitric oxide release in LPS-induced immune cells. It inhibits both the gene expression and function of IFN-β and IRF3, reduces iNOS gene expression, and disrupts the downstream signaling of the NF-κB pathway. Moreover, azamollugin inhibits two major pathways: the MyD88-dependent pathway, associated with the initial phase of inflammation, and the TRIF-dependent pathway, involved in the late phase of inflammation. In contrast, oxomollugin selectively inhibits the MyD88-dependent pathway, necessary for the initial phase of TLR4 signaling that induces NF-κB activation. By targeting both the MyD88 and TRIF pathways, azamollugin effectively suppresses NF-κB and IRF3 activation, demonstrating strong anti-inflammatory properties [77].

Despite its beneficial pharmacological activities, mollugin lacks water solubility, metabolic stability, and plasma stability, all of which continue to be major challenges for its clinical translation. To address these limitations, Hong et al. (2018) synthesized CF_3_-substituted mollugin derivatives with improved water and metabolic stability [78]. Among the synthesized mollugin derivatives, 2-Morpholinoethyl 6-hydroxy-2,2,8-trimethyl-2H-benzo[h]chromene-5-carboxylate demonstrates more effective anti-inflammatory activity in U937 and HT-29 colon cells compared to mollugin and mesalazine, along with enhanced water solubility and plasma and metabolic stability [78].

Further investigations on three mollugin-based compounds found that compound I, which is structurally modified with a distinctive pentacyclic ring containing a spirocyclic group, and compound II, which possesses a different four ring structure resulting from structural rearrangement, both have mollugin as their parent compound. Compound III is mollugin itself. The study was performed on three human cancer cell lines: A549, SGC-7901, and on HeLa cells. The results show that mollugin itself exhibits a weak cytotoxic impact on cell lines. The pentacyclic mollugin derivative shows a cytotoxic effect and, importantly, suppresses the NF-κB signaling pathway. Meanwhile, the structurally rearranged mollugin compound, referred to as compound II, exhibits a significant cytotoxic effect on the cancer cell lines and shows a synergistic effect on both the NF-κB and TNF-α signaling cascades [79]. In conclusion, the pharmacokinetics limitations can be managed by structural modifications, which address the challenges positioning mollugin as a promising therapeutic compound for treating various conditions. Future studies should aim to explore the dose-dependent relationships and safety margins of these derivatives to ensure their emergence as effective therapeutic candidates. The observed pharmacological effects and mechanisms of mollugin in different in vivo models is summarized in Table 1.

## 4. Pharmacokinetics and Toxicological Profile of Mollugin

In addition to numerous pharmacodynamic properties, pharmacokinetics is an important determinant in drug discovery and development and plays a major role in the future therapeutic usage of the drug. However, phytochemicals are often less studied for their pharmacokinetic profile over the pharmacodynamic effects. Likewise, mollugin has been investigated in only a few studies and the majority of the pharmacokinetic data are obtained from the in-silico predictions. These predictions reveal that mollugin exhibits a high absorption rate (around 95.8%), indicating better oral bioavailability. It also has poor water solubility and a low volume of distribution, indicating limited tissue distribution and significant binding to plasma proteins. Mollugin shows low permeability across the blood–brain barrier, restricting its diffusion into the brain.

According to pkCSM online predictions, https://biosig.lab.uq.edu.au/pkcsm/prediction (accessed on 25 July 2025), mollugin inhibits the CYP1A2 and CYP2C19 enzymes, indicating possible drug interactions with the drugs that use these enzymes for metabolism, therefore, further studies are warranted to establish the drug–drug interactions. Additionally, mollugin is neither hepatotoxic nor skin sensitizing. It does not inhibit hERG type I and II, indicating a lack of QT interval prolongation associated with ventricular arrythmia, and may therefor exhibit favorable cardioprotective properties. As the ADMET-predicted values of mollugin are within the accepted range for human use, it is expected to have good pharmacokinetic and pharmacodynamic properties

Many in vivo and in vitro studies have evaluated mollugin at different dose levels ranging from 5 mg/kg to 100 mg/kg, reporting therapeutic benefits and finding it unlikely to cause serious adverse events or mortality. No adverse events have been reported up to 100 mg/kg dose, which could be indicative of its relative safety in addition to its efficacy. Mollugin has been studied in various organ models and it exhibited organ protective properties. However, more experimental and regulatory toxicology studies, such as acute, subacute, and chronic toxicity studies, as well as carcinogenicity and genotoxicity studies, are still required to establish potent, safe, and effective dose for future investigation in human studies. The route of administration also needs to be established. In addition to efficacy, a favorable pharmacokinetic profile and assessment of drug–drug or food interactions are essential for the development of mollugin as phytopharmaceutical or nutraceutical for future use in humans. The pharmacokinetics and toxicological profile of mollugin are displayed in Table 2.

## 5. Discussion

Over the past few years, there has been a growing scientific interest in herbal drugs and they have gained much popularity, with increased focus on identifying the phytoconstituents contributing to their therapeutic properties. Though plant-based preparations are popularly used and show promise for therapeutic and preventive benefits, there is enormous focus on identifying the phytocompounds, attributing the biological actions, and elucidating the pharmacological and molecular mechanisms to rationalize their usage in therapeutics. To identify the safe and effective agents and promote the sensible usage of the plant-based medicines or utilization of bioactive phytocompounds, the pharmacological evaluation of phytocompounds derived from plants has gained momentum in the past decade [81].

Mollugin exhibits pleiotropic effects and poly pharmacological properties with potential actions on cell signaling pathways that play a major role in the onset and progression of oxidative stress, inflammation, apoptosis, and immune modulation in different diseases. When assessing the anti-inflammatory activity of mollugin in comparison with a well-explored natural bioactive like curcumin, mollugin shows overlapping effects like the suppression of the NF-κB and MAPK pathways [36,39,60]. Additionally, mollugin activates the Nrf2/Keap1 pathway, which confers cellular protection against the oxidative stress [39].

Recently, structural modifications have been made to improve the pharmacokinetic profile of mollugin and make it more druggable. These modifications, such as the synthesis of oxomollugin and CF_3_-substituted analogs, help mollugin overcome stability and ADMET-related limitations, thereby making it a better candidate for drug development. This gives mollugin an advantage over other phytochemicals by offering better bioavailability [78].

Currently, a specific therapeutic window for mollugin has not been established across different cell types. Available evidence indicates that mollugin exerts a dose-dependent response; a lower concentration around 20 µM remains non-cytotoxic in 3T3-L1 cells, whereas a higher dose of 40–60 µM causes apoptosis and reduces cell viability [65]. These findings indicate the dose-dependent selectivity of mollugin in different models, and additional investigations are required to establish the precise therapeutic window and safety margins across different disease models.

Mollugin has the potential to modulate the immune system by downregulating the TAK1-NF-κB and MAPK signaling pathways, which are responsible for triggering inflammation. It can also activate the Nrf2 pathway, which protects cells from damage. This dual mechanism of mollugin makes it effective in treating conditions like sepsis and other diseases where systemic infection and inflammation can spread throughout the body [39]. In comparison with other anti-inflammatory compounds such as resveratrol and berberine, mollugin acts through a particular mechanism by targeting ZFP61, which inhibits inflammasome activity and reduces inflammation by affecting caspase 8-mediated inflammasome activation. Mollugin promotes bone health by increasing bone formation via the activation of the BMP-2/p38/Smad pathway [52]. Furthermore, it impedes bone degradation by reducing the expression of c-Fos and NFATc1, two genes critical for osteoclastogenesis [53]. This unique characteristic makes mollugin more efficacious than other phytoconstituents such as genistein, which predominantly emphasize osteoblast activation. These make mollugin a promising candidate to treat conditions like osteoporosis, rheumatoid arthritis, and other bone-related diseases.

Mollugin has also exhibited potent anticancer activities. It has the potential to induce ferroptosis in colorectal cancer and hepatocellular carcinoma by elevating the levels of ROS and SOD, while downregulating GPX4, GSH, and IGF2BP3 [18,19,20,23,25]. Mollugin has a mechanism of action similar to derivatives of artemisinin, both of which induce oxidative cell death in malignant cells. Moreover, mollugin may serve as an appropriate adjuvant to current chemotherapeutic regimens, possibly resulting in enhanced synergistic effects. Its ability to suppress MDR1 allows mollugin to overcome multidrug resistance [26]. In neurodegenerative diseases, mollugin exhibits significant neuroprotection in endothelial and hippocampal experimental models through the regulation of the BDNF/TrkB and GLP-1R signaling pathways [9]. This indicates that mollugin could be a potential candidate for Alzheimer’s disease, as well as for the management of type 2 diabetes-related cognitive impairment [48].

Despite these broad spectral activities, the progression of mollugin as a therapeutic agent in clinical applications may encounter several challenges such as poor water solubility and limited plasma stability. Despite this, structural derivatives with improved pharmacokinetic characteristics, such as oxomollugin and azamollugin, are capable of overcoming these challenges. The intrinsic instability of many phytoconstituents brings complications in drug formulation, but its prolific ADMET properties make it a suitable choice for further study [74,77]. Future research should focus on nano-formulation development, toxicity evaluation, human in vitro studies, and phase 1 safety trials. Altogether, mollugin possesses a distinct pharmacological characteristic among other phytochemicals for managing complex diseases.

## 6. Conclusions and Future Remarks

Mollugin, a bioactive naphthoquinone derived from *Rubia cardifolia*, exhibits diverse pharmacological actions through the regulation of oxidative stress, apoptosis, inflammation, and immune response. The reported preclinical data support its antioxidant, anti-inflammatory, anticancer, antimicrobial, and neuroprotective effects, largely mediated through modulation of the MAPK, TAK1-NF-κB, Nrf2/Keap1, ferroptosis, BDNF/TrkB, and GLP-1R signaling pathways. Notably, most of these findings are focused on in vitro studies and in vivo findings are limited across several disease models, restricting the current understanding of their translational potential. Among the available evidence, anticancer and anti-inflammatory effects of mollugin are supported by strong preclinical data, while its osteogenic and neuroprotective activities are moderately supported. In contrast, its antimicrobial, gut-related, antiadipogenic, and pain-modulating effects are less established and require extensive validation in appropriate experimental models. Mollugin derivatives, such as oxomollugin and azamollugin, have demonstrated enhanced bioactive potential and an improved ADMET profile; however, further investigations are needed to validate their therapeutic properties.

Compared to well-studied phytoconstituents such as curcumin and resveratrol, mollugin demonstrates marked multifunctional activity, but the extensive investigation into its pharmacokinetic and safety profiles remains limited. Future studies should focus on addressing these research gaps through in-depth in vivo, toxicological, and formulation-based validation to enhance the ADMET profile. Establishing a precise therapeutic window and safety margin will be a critical finding in advancing mollugin from an experimental compound to a clinically relevant multitarget molecule.

## Figures and Tables

**Table 1:** The observed pharmacological effects and mechanisms in different in vivo models.

Type of Disease	Animal Model	Effects	Mechanism	Mollugin Dosage	Duration	Reference
Inflammation	Male mice, xylene-induced ear edema (20 μL)	Mollugin derivatives inhibit NF-κB transcription and reduce inflammation	Downregulates NF-κB signaling pathway, inhibits LPS-induced expression of p65	100 mg/kg, 0.1 mL/20 g body weight	24 h	[41]
Colorectal cancer	Male nude mice (5–6 weeks old), xenografted with DLD-1 cells (2 × 10^6^ cells in 200 μL PBS)	Limits proliferation, induces lipid peroxidation, and drives ferroptosis of CRC cells	↑ ROS, Fe^2+^, MDA ↓ IGF2BP3, GPX4, GSH	75 mg/kg once per day i.g, and/or i.p injected with Fer-1 (10 mg/kg, once every other day)	-	[18]
Allergic airway inflammation	6- to 8-week-old female C57BL/6 mice, induced with shrimp tropomyosin combined with Al (OH)_3_ (1.25 mg), i.p: 20 μg on days 0, 7, 14; intratracheal instillation: 40 μg on day 21	Reduces airway inflammation, mucus secretion, eosinophil infiltration, and Th2 cytokines (IL-4, IL-5, IL-13)	Targets p38 MAPK and PARP1 → inhibits M2 macrophage activation and IL-5 expression	5 mg/kg and 10 mg/kg i.p	From day 19 to 25	[40]
Diabetic cognitive dysfunction	8-week-old male C57BL/6 mice, injected STZ (60 mg/kg, i.p) for 3 consecutive days	Improves cognition, lowered ROS level, and apoptosis	Activates GLP-1R → cAMP/PKA pathway → ↑ Ca^2+^ influx, ↓ Pik3ca/Akt1/Mapk1	25, 50, and 100 mg/kg per day, p.o	6 weeks	[48]
Hepatocellular carcinoma	6- to 8-week-old BALB/c male mice, s.c injected 1.0 × 10^6^ cells per mouse on the right forelimb armpit	Inhibits HepG2 cell tumor growth by inducing DNA damage and S phase arrest	↑ ROS → DNA damage (↑ p-H2AX) → S phase cell cycle arrest (cyclin A2, CDK2) → tumor inhibition	25 mg/kg (low dose), 50 mg/kg (medium dose), 75 mg/kg (high dose), i.g	14 days	[19]
Ulcerative colitis	6- to 8-week-old C57BL/6 male mice, induced by administering 3% DSS solution as drinking water	Reduces weight loss, colon injury, and disease activity index	Downregulates IL-1β, TNF-α, IFN-γ, acts through TLR4-independent NF-κB suppression pathway	10, 20, 40 mg/kg, p.o	10 days	[37]
Klebsiella pneumonia	Sprague Dawley rats of either sex, induced by administering Klebsiella pneumoniae solution (0.1 mL), orotracheal incubation	Lowers WBC and PMN count in blood, reduces lung inflammation and bleeding, suppresses inflammatory cell infiltration and alveolar wall thickness	Inhibits NF-κB pathway ↓ inflammatory response Blocks MAPK pathway ↓ immune damage, regulates lung inflammation	80 mg/kg, p.o	7 days	[60]
Cervical cancer	BALB/c female athymic nude mice, s.c injected 0.2 mL HeLa cells (5 × 10^7^ cells/mL)	Suppresses tumor growth	Inhibits NF-κB activation, prevents p65 entry into nucleus and breakdown of IκBα, inhibits IKK phosphorylation, suppresses cancer gene expression	Given three times a week as an oral suspension of mollugin in saline at a dose of 25 and 75 mg/kg body weight	-	[25]
Bone loss/osteoclast-related disorder	ICR mice, induced with LPS (5 mg/kg, i.p) on day 1 and 4	Inhibits osteoclast differentiation, prevents LPS-induced bone loss, suppresses F-actin ring formation	↓ RANKL-induced phosphorylation of ERK, JNK, p38, AKT, GSK3β, ↓ c-FOS and NFATc1, ↓ OSCAR, TRAP, DC-STAMP, integrins, CtsK, reduces osteoclast activity and bone resorption	100 mg/kg, p.o	8 days	[53]

**Table 2:** Predicted and experimentally determined pharmacokinetic and toxicological parameters of mollugin.

Property	Predicted Value	Source
Intestinal absorption	93.697%	Predicted (pkCSM)
Volume of distribution	0.216 log L/kg	Predicted (pkCSM)
BBB permeability	0.541 log BB	Predicted (pkCSM)
CNS permeability	−1.844 log PS	Predicted (pkCSM)
Total clearance	0.629 log mL/min/kg	Predicted (pkCSM)
Maximum tolerated human dose	0.42 log mg/kg/day	Predicted (pkCSM)
LD_50_	2.459 mol/kg	Predicted (pkCSM)
LOAEL	2.11 log mg/kg_bw/day	Predicted (pkCSM)
C_max_	52.10 ± 6.71 ng/mL	Rodent experimental model [80]
T_max_	1.99 ± 0.21 h	Rodent experimental model [80]
t_1/2_	9.02 ± 2.14 h	Rodent experimental model [80]

## Data Availability

No new data were created in this study. Data sharing is not applicable to this article.
